# Etiologic Assessment of Parasitic Contaminants on Raw Vegetables Sold in Accra, Ghana

**DOI:** 10.1155/japr/1316135

**Published:** 2025-10-14

**Authors:** Reuben Essel Arhin, Henry Kwadwo Hackman, Charity Ahiabor, Henry Selasi Datsomor, Nana Kwame Ohene-Bekoe Junior, Rejoice Kafui Adjei, Siaw Teye Okpenor, Fauzia Awudu, Joellina Anang

**Affiliations:** ^1^Department of Science Laboratory Technology, Accra Technical University, Accra, Ghana; ^2^Department of Medical Laboratory Technology, Accra Technical University, Accra, Ghana

**Keywords:** etiologic assessment, foodborne diseases, Ghana, parasitic contaminants, raw vegetables, transmission

## Abstract

Parasitic infections can occur by eating contaminated vegetables. This study was aimed at assessing the risk of transmission of intestinal parasitic contaminants from raw vegetables sold in Accra. Three hundred (300) vegetables (cabbage, carrots, green pepper, tomatoes, lettuce, and cucumbers) were sampled from Accra markets, and survey data on potential risk factors were collected from vendors using a semistructured questionnaire. Sediments prepared from washings of the vegetables were used for wet mounts, and the parasites were identified using a microscope. The parasitic contamination rate was 55.0%. Hookworms (16.2%) and *Strongyloides stercoralis* (14.6%) were the predominant parasitic metazoans, whereas *Giardia* species (14.1%) was the predominant parasitic protozoan. There was a significant association between the category of vegetables (*p* = 0.0489) and parasitic contamination. There was also a significant association between the mode of display of the vegetables (*p* = 0.0334) and parasitic contamination. Parasitic contaminants were 1.393 times more likely to occur in green leafy vegetables than in others and 1.762 times more likely to occur on vegetables displayed on the floor than those in baskets. There is a high prevalence of parasitic contaminants on vegetables sold in Accra, and this is potentially a source of transmission of parasitic infections if proper decontamination procedures are not followed before consumption.

## 1. Introduction

Globally, parasitic infections are known to be a major public health problem [1], and intestinal parasitism accounts for an estimated 450 million deaths [2]. Approximately 2 billion people have been estimated to be infected with intestinal parasites [3], and this is associated with a high economic and mortality burden in developing countries [4]. Foodborne transmission of parasites is critical due to several factors that include the consumption of raw foods [3].

Parasitic infections can occur through the consumption of different kinds of food, and these include contaminated vegetables [5], which are often consumed as ready-to-eat salads. Parasitic contamination rates as high as 78.5% have been reported to occur on vegetables [6]. The transmission of protozoan cysts, oocysts, helminth eggs, and larvae has been reported to occur through the consumption of raw vegetables [7], and a significant association has been reported to exist between intestinal parasitic infections in people and the consumption of raw fruits and vegetables [8, 9]. This is because several kinds of food become contaminated as these move from the farm to the dining table during the preharvest, harvest, or postharvest stages [10, 11]. Therefore, parasitic contamination of raw vegetables is not only due to poor hygiene practices during food preparation but is also a result of the source of the food and the handling it undergoes before it gets to the consumer [12]. For this reason, the prevalence of pathogens, especially on vegetables that are consumed without thermal treatment, has public health implications [10].

Primarily, the use of sewage water for the irrigation of vegetable crops intended for human consumption can lead to contamination with parasitic helminths and protozoans [8, 13]. Secondly, poor management of human and animal excreta in the environment can also contribute to the contamination of vegetables from run-off surface water, which may flow into vegetable farms [11] or be used as untreated manure [14]. Also, flies may transfer parasites from human and animal excreta on their appendages onto vegetables [15] during their exploration and feeding activities. Furthermore, the personal hygiene practices of vegetable vendors may lead to the occurrence of parasitic contaminants on raw vegetables, key among which is hand hygiene. Although vegetables offer several health benefits, these come with an increased risk of acquiring foodborne diseases, especially when consumed raw or undercooked [16].

Globally, geohelminths such as *Ascaris lumbricoides*, *Trichuris trichiura*, and *Strongyloides stercoralis* are among the most predominant parasites [17] causing infections. However, *Cryptosporidium* spp., *Entamoeba histolytica*, and *Ascaris lumbricoides* are the main parasites reported to be transmitted through raw vegetables [18]. Parasitic protozoa on raw vegetables and fruits are known to be a significant contributor to foodborne diseases [17]. Common parasitic protozoans such as *Balantidium coli*, *Entamoeba coli*, and *Giardia duodenalis* can cause diarrhea [19]. Medical complications such as anemia, severe digestive disorders and abdominal pain, growth disorders in children, aggression, and weight loss, as well as physical and mental injuries [20] are caused by parasitic metazoans such as *Ascaris lumbricoides*, *Enterobius vermicularis*, hookworms, *Strongyloides stercoralis*, *Trichuris trichiura*, *Fasciola hepatica*, and *Taenia* spp. in about 3.5 billion people annually [19]. Further complications of parasitosis such as diarrhea and malabsorption, blood loss, impaired work capacity, and reduced growth rate are significant health and social challenges [21]. Therefore, endemic intestinal parasitic infections are closely associated with socioeconomic development processes in many countries, and the control of parasitic infections is a sociopolitical concern [21].

Gastroenterological diseases occur frequently in developing countries [22] such as Ghana. In the sub-Saharan Africa region, which includes Ghana, an estimated 250 million people have at least one intestinal parasite [2]. Despite concerns regarding the transmission of foodborne diseases through raw vegetables, in Ghana, little attention has been paid to parasites as most studies have focused on bacteria and fungi. Accra, the capital of Ghana, is a big city with a large population, and lots of food items cultivated within and outside the city are sold in open markets in the metropolis. There are many food services in the city, and most of these come along with menus which include vegetables grown inside or outside the city. This places the population at high risk of intestinal parasitic infections. However, there is a paucity of data on the etiology of parasitic contaminants on vegetables sold in Accra. This study was aimed at assessing the risk of transmission of intestinal parasitic contaminants from raw vegetables sold in Accra.

## 2. Materials and Methods

### 2.1. Study Design

A cross-sectional study was conducted in three open markets in Accra in August 2024. These are Madina Market, Adabraka Market, and Tudu Market. These markets are vending points for different kinds of vegetables received from various places in and outside the city.

### 2.2. Sample Collection

Samples were collected using a modification of the method described by [23]. A total of 300 fresh vegetables comprising 50 pieces each of cabbage, carrots, green pepper, tomatoes, lettuce, and cucumbers were purchased from 271 different vendors. Samples were collected in separate Ziploc bags and labeled for subsequent transport to the Accra Technical University Microbiology Laboratory.

### 2.3. Field Data

Verbal consent was obtained from the vegetable vendors, and a semistructured questionnaire was used to collect data on the sociodemographic and hygiene practices of the participating vegetable vendors as well as the vegetable exposure and handling processes.

### 2.4. Sample Processing

Large vegetables such as cabbage and lettuce were aseptically chopped and mixed to obtain a representative sample. From the mix, 10 g of the sample was weighed and transferred into a Ziploc bag. For smaller vegetables, whole vegetables were placed in Ziploc bags. A volume of 50 mL 0.85% NaCl solution was added to each sample in the Ziploc bag. This was sealed and vigorously agitated. Each resulting wash solution was transferred into a falcon tube and left in an upright position for 12 h for sedimentation of potential parasitic life forms [7]. The upper layer was decanted, and the remaining sample was centrifuged at 2000 rpm for 15 min. The supernatant was carefully decanted to leave behind a sediment.

### 2.5. Microscopic Examination

Each sediment was transferred to a fresh microscope glass slide using a sterile inoculating loop. This was examined as a saline wet mount under a light digital microscope using low-power and high-power magnifications to identify parasitic life forms. A second wet mount was prepared for microscopic examination using iodine solution. Parasitic protozoans and metazoans were identified with the aid of the CDC Laboratory Identification of Parasites of Public Health Concern manual [24]. Identified parasites were confirmed by a parasitologist.

### 2.6. Data Analysis

All data obtained were entered into Microsoft Office Excel and imported into STATA MP14 (Stata Corp LLC, College Station, Texas, United States) for descriptive and inferential analysis. Primarily, for each variable, the percentage frequency of the observations was found. Second, the chi-square test was used to determine the significance of the association between contamination status and risk assessment variables. Observations were summarized into 2 × 2 tables for the variables, sex (male and female), age (< 30 and ≥ 30 years), formal education (no and yes), market (Madina and others), fingernail status (trimmed and untrimmed), vegetable (green leafy and others), day of sampling (August 14 and August 16), washing status (washed and not washed), source of water (pipe borne and others), and mode of display (basket and floor) to allow for the determination of risk and odds ratios using the status “contaminated” and “not contaminated” as the case group and the control group, respectively. Furthermore, a multinomial logistic regression was performed to create a model of the relationship between the predictor variables and the contamination status of the vegetables (contaminated or not contaminated), and the data improved with the addition of the predictor variables. *p* < 0.05 was considered statistically significant.

## 3. Results

Most of the respondents (Table 1) were female vegetable vendors (98.7%), in their 20s (43.4%), had basic education (42%), and were engaged in their trade activities at the Madina (37.7%) and Tudu (38.0%) markets. Most of these vegetable vendors kept trimmed fingernails (86.3%).

Most of the vegetables (Table 2) on display at the markets were obtained from wholesale vendors (90.7%), and most were not washed prior to commercial display (86%). Pipe water (100%) was the source of water used to cleanse the vegetables that were reported to be washed (14%). Furthermore, most of the vegetables were displayed in baskets (72.7%) and sampled for processing in the afternoon (62.7%).

There was a 55.0% contamination rate of the samples of vegetables (Table 3). There was no significant association between the occurrence of parasitic contaminants and the sex of the study participants (*p* = 0.850), their age (*p* = 0.649), their level of education (*p* = 0.343), the market where they worked (*p* = 0.671), and the status of their fingernails (*p* = 0.121).

There was a significant association between parasitic contamination of the vegetables and the type of vegetable (*p* < 0.001) as well as the mode of display (*p* = 0.033) (Table 4). However, there was no significant association between the presence of parasitic contaminants and the day of sample collection (*p* = 0.658), the washing status (*p* = 0.889), and the period of sample collection (*p* = 0.671).

There was a significant association (*p* = 0.0489) between the category of vegetable and the presence of parasitic contaminants on the vegetables. The contamination was 1.393 times more likely to occur for green leafy vegetables than for other vegetables (Table 5). There was a significant association (*p* = 0.033) between the mode of display of the vegetable at the market and the presence of parasitic contaminants on the vegetables. The contamination was 1.7617 times more likely to occur for vegetables displayed on the floor than for those displayed in baskets.

Of 382 occurrences of parasites distributed on the fresh vegetables, 35.6% were due to parasitic protozoan cysts (Table 6). *Entamoeba* spp. had the least occurrence (5.0%) on vegetable samples. Other parasites identified were cystic stages of *Giardia* spp. (14.1%), *Balantidium* spp. (9.7%), and *Cryptosporidium* spp. (6.8%) for the protozoans. The vegetables that were most frequently contaminated with parasitic protozoans were lettuce (9.4%) and cabbage (7.3%).

Of 382 occurrences of parasites distributed on the fresh vegetables, 64.4% were due to parasitic metazoan eggs (Table 7). The most predominantly distributed parasites were the hookworms (16.2%), and these were mostly found on tomatoes (5.2%) and lettuce (3.4%). Other parasites identified were *Strongyloides stercoralis* (14.6%), *Ascaris lumbricoides* (12.8%), *Trichuris trichiura* (11.8%), and *Taenia* spp. (9.2%). Cabbage (12.8%) and tomatoes (12.6%) were the most frequently contaminated with parasitic metazoans.

The fit between the model containing only the intercept and data improved with the addition of the predictor variables (display, type of vegetable, and fingernail status of vendor), *χ*^2^(3, *N* = 300) = 26.79, *p*seudo *R*^2^ = 0.0648, *p* < 0.001 (Table 8). However, in the model, the only significant association was the category of vegetable (*p* < 0.01). The category of vegetable increased the risk of contamination and decreased the risk of the vegetable not being contaminated (RRR > 1).

## 4. Discussion

This study was aimed at assessing the risk of transmission of intestinal parasitic contaminants on raw vegetables.

Primarily, there was a high parasite contamination rate of the vegetables. A contamination rate of 1.9%–9.3% for fruits and vegetables has been previously reported to be a significant underestimate, especially in countries with poor hygiene [17]. Ezatpour et al. reported a contamination rate of 52.7% in Iran [7]. Hajipour et al. reported that 53.14% of vegetables from fields and 18.23% of ready-to-eat vegetables from greengrocers and markets in Iran had parasitic contaminants [25]. Falcone et al. in Argentina reported a rate of 58.6% for leafy vegetables in farms [18], Gabre and Shakir reported a rate of 46% in Saudi Arabia [12], Nahhas and Aboualchamat in Syria reported a rate of 34.4% [22], and Said in Egypt reported a rate of 31.7% [26]. However, contamination rates as low as 12.5% have been reported by M'rad et al. for vegetables in retail markets in Tunisia [27]. High contamination rates may potentially contribute to a high prevalence of parasitic infections. In countries such as Yemen, intestinal parasitic infection as high as 54.1% has been reported to occur among school children [1].

Secondly, the contaminants comprised a diversity of parasites associated with infections in different study settings. More than 100 parasites are known to be a matter of public health concern [28]. Protozoa cysts and oocysts such as *Entamoeba* sp., *Giardia* sp., eggs of *Ascaris lumbricoides*, *Taenia* sp., and hookworms have been associated with vegetable-borne outbreaks [29, 30]. Ezatpour et al. have reported the presence of parasitic contaminants such as *Ascaris lumbricoides* eggs, *Enterobius vermicularis* eggs, *Giardia* spp. cysts, and *Strongyloides stercoralis* eggs, among others, on raw vegetables [7]. Fallah et al. have also reported *Ascaris lumbricoides* eggs (14.1%), *Taenia* spp. eggs (9.2%), *Toxocara* spp. eggs (3.3%), and *Giardia* spp. cysts (8.2%), among others, on raw unwashed vegetables in Iran [29]. Some, like *Cryptosporidium* spp. and *Giardia duodenalis*, are foodborne protozoan parasites of public health concern that have been linked to numerous outbreaks [31]. These were among the parasites identified in this study. This suggests that there is a high risk involved in consuming these vegetables if improperly handled and underprocessed; hence, it emphasizes the importance of decontaminating vegetables before consumption.

Despite some vendors claiming to wash the vegetables with pipe-borne water before displaying these for sale, washing status was not associated with the occurrence of parasitic contaminants. This could be due to contamination of the pipe-borne water, which may occur from time to time due to leakages, or postwashing contamination of the vegetables while on display for sale. Also, at the sales point, there is the tendency to touch or handle the vegetables by both customers and vendors. These factors may potentially account for the observation. Since prewashing procedures do not guarantee the elimination of parasites from salad vegetables [30], proper washing before consumption is essential to reduce the potential for transmission of parasitic infections [29].

Furthermore, in this study, the only significant associations with contamination were the type of vegetable and the mode of display. In consensus, it has been previously reported that the environment in which vegetables are sold, as well as the vegetables, is a potential source of microbial contamination [23]. In contrast, Hassan et al. did not find any significant association between the type of vegetable and the presence of parasitic contaminations [13]. Again, in contrast, Al-Mekhlafi et al. have reported that illiteracy and not keeping trimmed fingernails are significantly associated with higher rates of parasitic infections in family circles [1], but these were not found to be factors associated with the contamination of the vegetables in this study. Socioecological factors such as the behaviors of people and their interactions with specific places increase the risk of parasitic infections [28]. Among other factors, food is an essential part of the human socioecology, especially in the market environment; hence, several variables were expected to be associated with parasitic contamination of the vegetables but were not.

Despite these factors, the green leafy vegetables like lettuce and cabbage were the most predominantly contaminated. Similarly, some previous studies have reported that leafy vegetables such as lettuce and/or cabbage have the highest prevalence and concentrations of parasites [3, 4, 23]. The contamination of leafy vegetables has been attributed to their low-lying form and rough physiology, which supports the retention of parasites during washing [23]. Raw vegetables can also become contaminated with parasites and, therefore, are a source of transmission of protozoan cysts, helminth eggs, and infectious larvae [10]. Ezatpour et al. reported that among leek, green onion, mint, radish, and garden cress, the least contaminated was green onion (34.5%) during spring and garden cress (10.9%) during winter [7]. The least contaminated vegetable in this study was green pepper. Although different vegetables were studied, differences in the prevalence of the various parasitic contaminants between this and other studies may be influenced by factors such as differences in the sensitivity and specificity of the detection methods [16].

Finally, it has been reported in a review study by Amahmid et al. that *Ascaris* sp. and *Giardia* sp. are the predominant parasites on vegetables in several countries [3]. However, other geohelminths, specifically hookworms and *Strongyloides stercoralis*, were the predominant parasitic contaminants in this study. The detection of helminth eggs on vegetables is mainly associated with soil contamination rather than contamination from irrigation water [26]. Since the distribution of intestinal parasites depends on geographic and socioenvironmental factors, climatic variables and soil characteristics determine the viability of parasitic forms [18]; hence, variations can be expected in samples from different areas. Kame-Ngasse et al. have previously reported the predominance of *Strongyloides stercoralis* on raw vegetables [23]. The predominance of the geohelminths in this study suggests that contact with the soil due to unhygienic handling practices may be a significant risk factor. According to the WHO, hookworm infection is among the 10 most common infections in the world, and strongyloidiasis is of local or regional public health concern [21]. Approximately 613.9 million people around the world have strongyloidiasis and are at risk of the fatal hyperinfection syndrome, with a fatality rate of 15%–87% [20]. *Cryptosporidium* spp., *Entamoeba histolytica*, and *Giardia lamblia* are the most prevalent intestinal protozoa infecting humans [16], and these species were identified as parasitic contaminants in this study.

One limitation of the study was the difficulty in trying to obtain responses to some of the risk assessment queries since the study participants were evasive when it came to answering some questions. A larger number of responses may have influenced the outcome of the study.

## 5. Conclusions

Raw vegetables sold in Accra are a potential public health risk due to the presence of parasitic contaminants associated with medical complications. Strong measures should be put in place to reduce the presence of these parasitic contaminants at the preharvest, harvest, postharvest, and vending points. These should include regulations to check the source of water used for production, the use of manure, and proper sewage management in the farming areas, and hygienic handling from the farm to the vending points. At the vending point, authorities should enforce a ban on the display of raw vegetables on the floor, and there should be education to inform both the vendors and public on the need to keep vegetables clean as well as to decontaminate vegetables before consumption, especially in cases where they are to be consumed raw. These measures will help reduce the potential risk of transmission of parasitic contaminants from raw vegetables to consumers and safeguard the health of the public.

## Figures and Tables

**Table 1:** Sociodemographic characteristics and personal hygiene of the vegetable vendors.

**Variable**	**Observations**	**Frequency (%)**
Sex	Male	4 (1.3)
	Female	296 (98.7)
	Total	300 (100.0)

Age	< 20	3 (5.7)
	20s	23 (43.4)
	30s	20 (37.7)
	40s	7 (13.2)
	Total	53 (100)

Education level	None	14 (28)
	Basic	21 (42)
	Secondary	14 (28)
	Tertiary	1 (2)
	Total	50 (100)

Market	Madina	113 (37.7)
	Adabraka	73 (24.3)
	Tudu	114 (38.0)
	Total	300 (100.0)

Fingernail status	Trimmed	259 (86.3)
	Untrimmed	41 (13.7)
	Total	300 (100)

**Table 2:** Vegetable handling practices and processes.

**Variable**	**Observations**	**Frequency (%)**
Source of sample	Wholesale vendors	271 (90.7)
	Retail vendors	29 (9.3)
	Total	300 (100.0)

Washing status	Washed	41 (13.7)
	Not washed	259 (86.3)
	Total	300 (100.0)

Source of wash water	Pipe borne	25 (100)
	Others	0 (0.0)
	Total	25 (100)

Mode of display	On floor	82 (27.3)
	In basket	218 (72.7)
	Total	300 (100.0)

Period of sampling	Morning (before 12)	112 (37.3)
	Afternoon (after 12)	188 (62.7)
	Total	300 (100.0)

**Table 3:** Distribution of contaminated vegetables by sociodemographics and personal hygiene of vendors.

**Variable**	**Parasitological status (%)**		**p** **value**
	**Contaminated**	**Not contaminated**	
Sex			0.850
Female	163 (54.3)	133 (44.3)	
Male	2 (0.7)	2 (0.7)	
Total	165 (55.0)	135 (45.0)	
Age			0.649
20s	10 (18.9)	12 (22.6)	
30s	10 (18.9)	10 (18.9)	
40s	5 (9.4)	2 (0.7)	
< 20	1 (1.9)	1 (1.9)	
Total	26 (49.1)	27 (50.9)	
Education level of vendors			0.343
None	9 (18.0)	5 (10.0)	
Basic	8 (16.0)	13 (26.0)	
Secondary	7 (14.0)	7 (14.0)	
Tertiary	1 (2.0)	0 (0.0)	
Total	25 (50.0)	25 (50.0)	
Market			0.671
Madina	64 (21.3)	49 (16.3)	
Adabraka	42 (14.0)	31 (10.3)	
Tudu	59 (19.7)	55 (18.3)	
Total	165 (55.0)	135 (44.9)	
Fingernail status of vendors			0.121
Trimmed	138 (46.0)	121 (40.3)	
Untrimmed	27 (9.0)	14 (4.7)	
Total	165 (100.0)	135 (100.0)	

**Table 4:** Distribution of contaminated vegetables by handling practices and processes.

**Variable**	**Parasitological status (%)**		**p** **value**
	**Contaminated**	**Not contaminated**	
Vegetables			< 0.001
Cabbage	36 (12.0)	14 (4.7)	
Carrot	18 (6.0)	32 (10.7)	
Cucumber	32 (10.7)	18 (6.0)	
Green pepper	13 (4.3)	37 (12.3)	
Lettuce	27 (9.0)	23 (7.7)	
Tomato	39 (13.0)	11 (3.7)	
Total	165 (55.0)	135 (45.0)	
Source of vegetables			0.400
Wholesale vendors	18 (6.0)	11 (3.7)	
Retail vendors	89 (29.7)	125 (41.7)	
Total	164 (54.7)	136 (45.3)	
Day of sample collection			0.658
August 14	64 (21.3)	49 (16.3)	
August 16	101 (33.7)	86 (28.7)	
Total	165 (55.0)	135 (45.0)	
Washing status			
Washed	117 (39.0)	19 (6.3)	0.889
Not washed	142 (47.3)	22 (7.3)	
Total	259 (86.3)	41 (13.7)	
Source of wash water			
Pipe borne	14 (4.7)	11 (3.7)	
Other	0 (0.0)	0 (0.0)	
Total	14 (56.0)	11 (44.0)	
Mode of display			0.033
In basket	111 (37.0)	107 (35.7)	
On floor	53 (17.7)	29 (9.7)	
Total	165 (55.0)	135 (45.0)	
Sampling time			0.671
Morning	49 (16.3)	63 (21.0)	
Afternoon	87 (29.0)	101 (33.7)	
Total	136 (45.3)	164 (54.7)	

**Table 5:** Risk assessment for presence of parasitic contaminants.

**Variable (observations)**	**Risk ratio**	**Odds ratio**	**Pr** > **χ**^2^
Sex (male and female)	1.101	1.226	0.8540
Age (< 30 and ≥ 30 years)	0.863	0.739	0.5315
Formal education (no and yes)	1.446	2.250	0.2077
Market (Madina and others)	1.017	1.040	0.8692
Fingernail status (trimmed and untrimmed)	0.809	0.591	0.121
Category of vegetable (green leafy and others)	1.393	1.636	0.0489
Source (wholesale vendors and retail vendors)	1.493	2.298	0.4000
Day of sampling (Aug. 14 and Aug. 16)	1.049	1.112	0.6577
Washing status (washed and not washed)	0.994	0.954	0.8890
Source of water (pipe borne and other)	—	—	—
Mode of display (basket and floor)	1.269	1.7617	0.0334
Sampling period (morning and afternoon)	0.945	0.903	0.6707

**Table 6:** Prevalence and distribution of parasitic protozoan contaminants in raw vegetables.

**Vegetables**	**Parasitic protozoans (%)**				**Total**
	***Giardia* spp.**	***Entamoeba* spp.**	***Balantidium* spp.**	***Cryptosporidium* spp.**	
Lettuce	13 (3.4)	4 (1.1)	11 (2.9)	8 (2.1)	36 (9.4)
Carrot	8 (2.1)	3 (0.8)	7 (18.9)	4 (1.1)	22 (5.8)
Green pepper	7 (1.8)	3 (0.8)	4 (1.1)	3 (0.8)	17 (4.5)
Cucumber	6 (1.6)	2 (0.5)	3 (0.8)	3 (0.8)	14 (3.7)
Tomato	9 (2.4)	3 (0.8)	5 (1.3)	2 (0.5)	19 (5.0)
Cabbage	11 (2.9)	4 (1.1)	7 (1.8)	6 (1.6)	28 (7.3)
Total	54 (14.1)	19 (5.0)	37 (9.7)	26 (6.8)	136 (35.6)

**Table 7:** Prevalence and distribution of parasitic metazoan contaminants in raw vegetables.

**Vegetables**	**Parasitic metazoans (%)**					**Total**
	***Ascaris lumbricoides***	**Hookworms**	***Strongyloides stercoralis***	***Taenia* spp.**	***Trichuris trichiura***	
Lettuce	4 (1.1)	13 (3.4)	11 (2.9)	8 (2.1)	7 (1.8)	43 (11.3)
Carrot	3 (0.8)	8 (2.1)	7 (1.8)	4 (1.1)	12 (3.1)	34 (8.9)
Green pepper	3 (0.8)	7 (1.8)	4 (1.1)	3 (0.8)	8 (2.1)	25 (6.6)
Cucumber	15 (3.9)	3 (0.8)	17 (4.5)	10 (2.6)	2 (0.5)	47(12.3)
Tomato	12 (3.1)	20 (5.2)	1 (0.3)	10 (2.6)	5 (1.3)	48 (12.6)
Cabbage	12 (3.1)	11 (2.9)	16 (4.2)	0 (0.0)	10 (2.6)	49 (12.8)
Total	49 (12.8)	62 (16.2)	56 (14.6)	35 (9.2)	44 (11.5)	246 (64.4)

**Table 8:** Multinomial logistic regression analysis of some risk factors.

**Contaminated**	**RRR**	**Standard deviation**	**Z**	**P** > |**Z**|	**95% Conf.**	**Interval**
Category of vegetable	1.378422	0.1013519	4.36	~0.000	1.193426	1.592095
Mode of display	1.51852	0.4245465	1.49	0.1355	0.8778941	2.626629
Fingernail status	0.6137535	0.2275735	−1.32	0.188	0.2967408	1.269436
	0.5453341	0.2340155	−1.41	0.158	0.2351756	1.264541

Abbreviation: RRR, relative risk ratio.

## Data Availability

The data is available on request.
